# Identification of Somatic Mitochondrial DNA Mutations, Heteroplasmy, and Increased Levels of Catenanes in Tumor Specimens Obtained from Three Endometrial Cancer Patients

**DOI:** 10.3390/life12040562

**Published:** 2022-04-09

**Authors:** Matthew J. Young, Ravi Sachidanandam, Dale B. Hales, Laurent Brard, Kathy Robinson, Md. Mostafijur Rahman, Pabitra Khadka, Kathleen Groesch, Carolyn K. J. Young

**Affiliations:** 1 Department of Biochemistry & Molecular Biology, Southern Illinois University School of Medicine, Carbondale, IL 62901, USA; dhales@siumed.edu (D.B.H.); mdmostafijur.rahman@siu.edu (M.M.R.); pabitra.khadka@siu.edu (P.K.); carolyn.young@siu.edu (C.K.J.Y.); 2 Simmons Cancer Institute, Southern Illinois University School of Medicine, Springfield, IL 62702, USA; lbrard@siumed.edu (L.B.); krobinson@siumed.edu (K.R.); 3 Department of Oncological Sciences, Icahn School of Medicine at Mount Sinai, New York, NY 10029, USA; ravi.mssm@gmail.com; 4 Department of Physiology, Southern Illinois University School of Medicine, Carbondale, IL 62901, USA; 5 Department of Obstetrics & Gynecology, Southern Illinois University School of Medicine, Springfield, IL 62702, USA; kgroesch@siumed.edu; 6 Division of Hematology/Oncology, Department of Internal Medicine, Southern Illinois University, Springfield, IL 62702, USA; 7 Center for Clinical Research, Southern Illinois University School of Medicine, Springfield, IL 62702, USA

**Keywords:** uterine cancer, endometrial cancer, human mitochondrial DNA (mtDNA) maintenance, homoplasmy, heteroplasmy, somatic mutations, next-generation sequencing (NGS), mtDNA copy number, mtDNA content, mtDNA topoisomers

## Abstract

Endometrial carcinoma (EC) is the most common type of gynecologic malignant epithelial tumor, with the death rate from this disease doubling over the past 20 years. Mitochondria provide cancer cells with necessary anabolic building blocks such as amino acids, lipids, and nucleotides, and EC samples have been shown to increase mitochondrial biogenesis. In cancer, mitochondrial DNA (mtDNA) heteroplasmy studies suggest that heteroplasmic variants encode predicted pathogenic proteins. We investigated the mtDNA genotypes within peri-normal and tumor specimens obtained from three individuals diagnosed with EC. DNA extracts from peri-normal and tumor tissues were used for mtDNA-specific next-generation sequencing and analyses of mtDNA content and topoisomers. The three tumors harbor heteroplasmic somatic mutations, and at least one mutation in each carcinoma is predicted to deleteriously alter a mtDNA-encoded protein. Somatic heteroplasmy linked to two mtDNA tRNA genes was found in separate tumors, and two heteroplasmic non-coding variants were identified in a single EC tumor. While two tumors had altered mtDNA content, all three displayed increased mtDNA catenanes. Our findings support that EC cells require wild-type mtDNA, but heteroplasmic mutations may alter mitochondrial metabolism to help promote cancer cell growth and proliferation.

## 1. Introduction

Endometrial carcinoma (EC) is the most common type of gynecologic malignant epithelial tumor, with the death rate from this disease increasing more than 100% over the past 20 years [1]. According to recent estimates, there were ~90,000 deaths and ~382,000 new cases of EC in 2018 [2]. EC is a devastating disease that results from the uncontrolled growth of cells within the endometrium; the inner layer, or mucosal lining, of the mammalian uterus that is comprised of an epithelial layer, glands, connective tissue (stroma), and blood vessels [3]. Endometrial tissue is responsive to hormones, and EC is thought to arise from estrogen stimulation that is unopposed by progestins. ECs are generally classified into two types. Type I endometrioid adenocarcinoma is the most common, representing more than 80% of EC cases, and is associated with unopposed estrogen stimulation. Type I ECs are generally low-grade tumors that exhibit glandular differentiation and likely originate from glandular cells [4]. Type II accounts for 10% of ECs and is associated with 40% of related deaths. Type II ECs are of papillary serous or clear cell histology and are typically high-grade tumors [1,5].

Many cancers display aerobic glycolysis, also known as the Warburg effect [6]. However, our current understanding is that increased glucose uptake and fermentation of glucose to lactate occur in cancer cells with functional mitochondria, and mitochondrial metabolism is essential for tumorigenesis [7,8]. The current evidence supports that cancer cells alter their mitochondrial functions to promote tumor growth [8]. In cancer cells, mitochondria provide the necessary building blocks such as amino acids, lipids, and nucleotides that are required for anabolism. Mitochondrial function in EC is essential as type I EC (estrogen-dependent) patient samples demonstrate increased mitochondrial biogenesis compared to matched hyperplasia samples [9]. Estrogen-mediated reactive oxygen species (ROS) production has been proposed to cause mitochondrial DNA (mtDNA) mutation [10].

Further, evidence supports that estrogen metabolites react with DNA and could cause mutations responsible for cancer initiation [11]. Large-scale next-generation sequencing (NGS) efforts support that the majority of all solid tumors harbor at least one mtDNA mutation [12]. Studies have also demonstrated that tumors acquire deleterious mtDNA coding mutations at a lower frequency than other mutations, supporting the role of functional mitochondria in tumor cells [13,14]. The multi-copied mitochondrial genome harbors 37 open reading frames required to support mitochondrial oxidative phosphorylation (OXPHOS). The necessity for our cells and tissue to maintain mtDNA is underscored by observations that mitochondrial disease mutations occur in all 37 mtDNA genes, and damage to mtDNA induces apoptosis [15,16]. Furthermore, genetic knockouts of genes encoding factors required to replicate mtDNA are embryonic lethal in mice [17]. Thus, mtDNA maintenance is needed to preserve OXPHOS function (and, by extension, cellular ATP production) that is required for EC cell growth and tumorigenesis.

A single cell can contain several thousand copies of mtDNA, which are distributed within hundreds of individual mitochondria or throughout an elaborate mitochondrial reticular network [18]. Human cancer cells have been reported to harbor clonal synonymous and non-synonymous mtDNA variants, so-called homoplasmic substitutions or homoplasmy. Additionally, cancer cells have been reported to contain heteroplasmic mixtures of wild-type (WT) and mutant mtDNA genotypes, known as mtDNA heteroplasmy [19,20]. Cytoplasmic hybrid (cybrid) experiments support mtDNA mutations driving cancer progression. A cybrid is formed by fusing an enucleated donor cell’s cytoplasm (containing mtDNA) with a recipient cell lacking mtDNA. A recipient nuclear genetic background cell lacking mtDNA is referred to as a rho zero cell (ρ^0^), while a donor cell lacking a nucleus but containing mitochondria is referred to as a cytoplast. Enucleated donor cytoplasts can be generated utilizing actinomycin D, which interferes with nuclear DNA (nDNA) replication [21]. Recipient cells lacking mtDNA can sometimes be selected when treated with low concentrations of ethidium bromide (EtBr) to inhibit mtDNA replication. However, the ability of a cell line to lose its mtDNA and maintain growth is rare [21,22,23,24,25]. Cybrids such as these were generated in a study utilizing a high metastatic potential (HMP) mouse tumor cell line and a low metastatic potential (LMP) mouse cell line. Experiments were conducted using the HMP cell line as the donor and the LMP cell line as the nuclear ρ^0^ recipient and vice versa. Intriguingly, cybrids with LMP nuclei and HMP mtDNA acquired metastatic transformation, whereas cybrids with HMP nuclei and LMP mtDNA lost metastatic potential [26]. Other studies have demonstrated similar findings, such as a non-tumorigenic cell having an enhanced tumorigenic phenotype when combined with tumor cell mitochondria [27].

A recent study lends support for heteroplasmic mtDNA mutations tuning cellular metabolism. The study used isogenic human cell lines harboring different heteroplasmic levels of a well-known mtDNA T8993G mutation that changed a crucial residue in the OXPHOS complex V ATP synthase subunit six gene, *ATP6*. A mitochondrially targeted zinc-finger nuclease was exploited to generate different levels of defined and stable T8993G heteroplasmy in three isogenic cell lines, low (7%), medium (45%), or high (80%). In cells with high levels of *ATP6* heteroplasmy, utilization of NADH by the OXPHOS machinery was altered, causing a cytosolic reductive carboxylation of glutamine and increased glycolysis and cell migration [28].

Interestingly, experiments utilizing human cancer cell lines have demonstrated that mtDNA heteroplasmy can be stably maintained after many passages in cell culture and even when cultures are derived from a single cell [19,29]. As the levels of heteroplasmy remain fixed during cell line passage (i.e., the variants do not clonally expand), these results suggest that specific cancer mtDNA alleles are not subjected to random genetic drift. Recently, we utilized the mtDNA-specific NGS (Mseek) approach to investigate the mtDNA genome of two human cell lines, HepaRG (hepatoma-derived) and SJCRH30 (rhabdomyosarcoma-derived). Mseek exploits mtDNA circular topology and treatment of DNA samples with exonuclease V (RecBCD) to specifically reduce the amount of nuclear genomic reads [19]. Nuclear-localized mtDNA pseudogenes or NUclear MiTochondrial sequences (NUMTs) contribute to errors in determining mtDNA heteroplasmy [30]. Exonuclease V cleaves linear double-stranded (ds) DNA in the 5′ to 3′ and 3′ to 5′ directions; therefore, circular mtDNA remains protected, while NUMTs are eliminated by digesting ds-nDNA. Using the Mseek approach, NGS analysis is simplified, and spurious NUMT signals are removed. Maintenance of HepaRG mtDNA heteroplasmy during growth in tissue culture was confirmed by sequencing DNA samples obtained from two different passages, passage 11 (41% C315CC and 37% G13633A) and 16 (42% C315CC and 33% G13633A). The G13633A/Gly433Ser substitution alters a conserved codon in the NADH dehydrogenase subunit 5 (*ND5*) gene predicted to alter mitochondrial function. Additional studies of mtDNA heteroplasmy in cancer specimens suggest that substitutions associated with heteroplasmy encode predicted pathogenic substitutions or protein truncations [13,14,31].

Suppose cancer cell heteroplasmic mtDNA mutations are detrimental, why are they not removed by purifying (negative) selection, and is there some selective advantage to a cancer cell maintaining mtDNA heteroplasmy? We hypothesize that cancer cells benefit from heteroplasmic mtDNA variants that alter mitochondrial metabolism to favor growth and proliferation. In this scenario, the mitochondrion would be under selective pressure to sustain a capacity of WT mtDNA molecules and, by extension, WT mtDNA-encoded OXPHOS subunits. To initially test this idea, we obtained peri-normal and tumor specimens from three individuals diagnosed with EC and performed Mseek NGS to identify somatic mtDNA mutations and heteroplasmy in tumors. Additionally, tumor and peri-normal tissue mtDNA content and topoisomers were analyzed.

## 2. Materials and Methods

### 2.1. Patients and Tissue Specimens

Peri-normal and tumor specimens were obtained from the Simmons Cancer Institute (SCI) at Southern Illinois University School of Medicine (SIUSOM) Tissue Bank. The surgically resected samples were de-identified to safeguard all aspects of patient care and privacy. The Springfield Committee for Research Involving Human Subjects (SCRIHS) is the institutional review board (IRB) at SIUSOM and approved the protocol (#12-177) under which the Tissue Bank facility operates. The Tissue Bank is authorized to obtain tissues for research that would otherwise be discarded following pathologic examination. Informed consent was obtained from all subjects prior to surgery and collection of tissue from the pathology laboratory. Upon collection, all tissue was wrapped in aluminum foil and immersed in liquid nitrogen while in the pathology laboratory, and subsequently stored in a liquid nitrogen freezer. Every specimen received from pathology was cut into three segments, with the middle section being the quality control (QC) block from which the QC slides were cut. Therefore, each QC slide is a good representation of its matched frozen specimen. Different pathologists evaluated each of the three sets of tumor and peri-normal samples.

Patient 1, a 75-year-old woman, was diagnosed with uterine carcinosarcoma. She underwent a hysterectomy, bilateral salpingo-oophorectomy, and staging. The mass infiltrated deeply into the myometrium wall to a subserosal location. Representative sections of peri-normal uterine and malignant uterine tumor tissues were submitted to the Tissue Bank. The tumor and peri-normal specimens on the QC slides were 100% and 0% tumors, respectively. The cancer was FIGO stage IB.

Patient 2, a 46-year-old woman, was diagnosed with endometrial adenocarcinoma, endometrioid type. She underwent a hysterectomy, bilateral salpingo-oophorectomy, and staging. The average myometrial thickness was 2.5 cm, and the tumor grossly invaded the superficial myometrium up to a depth of 0.5 cm. Representative sections of peri-normal uterine and malignant uterine tumor tissues were submitted to the Tissue Bank. Depending on the tumor specimens evaluated on the QC slides, tumor tissues a and b were 100% tumor, tumor tissues c and d were 90% tumor and 10% necrotic, and the perinormal sample was 0% tumor. The cancer was grade 1 and FIGO stage IA.

Patient 3, a 70-year-old woman, was diagnosed with invasive endometrial adenocarcinoma (endometrioid type), invading into the outer half of the myometrial wall. Two of the 18 lymph nodes were positive for metastatic adenocarcinoma. She underwent a hysterectomy, bilateral salpingo-oophorectomy, and staging. The uterus specimen was dissected to reveal an irregular tan endometrium on the right half of the cavity. Sections revealed tumor invading into the myometrium on the right side of the specimen. The uninvolved myometrium was 1 cm thick. Representative sections of peri-normal (fibromuscular stroma) uterine and malignant uterine tumor tissues were submitted to the Tissue Bank. On the QC slides, the tumor specimen was judged to be 90% tumor and 10% fibrosis, while the peri-normal sample was 0% tumor. The tumor was poorly differentiated, grade 3 and FIGO stage IIIC1.

### 2.2. DNA Extraction from Normal and Tumor Tissue

DNA extracts were prepared using a modification of the protocol described in Young et al. [32]. Briefly, 1–5 mm^3^ of tissue from a flash-frozen malignant tumor or peri-normal sample was resected. The tissue was cut on a pre-cooled glass plate sitting on ice then added to a 1 mL glass homogenizer containing 0.5 mL proteinase K digestion buffer, PKDB (100 mM Tris-Cl pH 8.5, 5 mM EDTA, 0.2% SDS, 200 mM NaCl, 0.3 mg/mL proteinase K, 1.1 mM 2-mercaptoethanol). The tissue was homogenized lightly with 20 passes of a loose-fitting pestle on ice, and then the homogenate was transferred to a sterile 1.5 mL microcentrifuge tube kept on ice. The homogenizer was rinsed with another 0.5 mL of PKDB, the homogenization procedure was repeated, and the second homogenate was combined with the first. The homogenate was digested overnight at 55 °C in reduced light. The next morning, the homogenate volume was measured with a micropipette, and fresh PKDB was added to generate a final volume of 1.02 mL. The sample was gently mixed and incubated for 1 h at 55 °C. The sample lysate was equally distributed between two microcentrifuge tubes such that each contained 0.51 mL. Cellular protein was removed by adding 0.17 mL of 5M NaCl to each microcentrifuge tube, the samples were gently mixed for 5 min, and the cellular protein was pelleted by centrifugation at 15,000× *g* for 15 min at 4 °C. The supernatants were collected into sterile 2 mL microcentrifuge tubes. In each tube, total nucleic acid plus 1 μL GlycoBlue co-precipitate (Invitrogen^TM^ a subsidiary of Thermo Fisher Scientific, Waltham, MA, USA) was precipitated with 1 mL of ice-cold 100% ethanol followed by centrifugation at 15,000× *g* for 15 min at 4 °C. The supernatant was carefully decanted, and pellets were washed with ice-cold 70% ethanol. The pellets were dried away from light for ~1 h then resuspended in 50 μL 1× TE buffer (10 mM Tris-Cl, pH 8.0, 1 mM EDTA) plus 1 mM dimethyl urea and 200 μg/mL RNase A (Thermo Fisher Scientific, Waltham, MA, USA). Samples were stored in the absence of light at room temperature overnight then frozen at −20 °C. According to the manufacturer’s specifications, DNA concentrations were measured using a Qubit fluorometer (Thermo Fisher Scientific).

### 2.3. mtDNA Next-Generation Sequencing and Data Analysis

The Mseek method of sample processing and deep sequencing of mtDNA, and the procedure for data analysis, were conducted as previously reported with slight modifications [19]. Briefly, Mseek consists of (i) digesting linear nuclear DNA (nDNA) with the ATP-dependent linear double-stranded DNA exonuclease V (ExoV), (ii) purifying the products using Ampure beads to remove short fragments, (iii) testing the results of the digestion with PCR primers specific for mtDNA and nDNA using 1 μL of the digested sample, (iv) Tagmentation, using transposase-based Nextera kit from Illumina, creates fragments, (v) amplifying the library utilizing universal adapters, (vi) size selection using a double-sided bead purification system to select ~350 bp sizes, and (vii) loading samples onto the Illumina NextSeq 500 platform (Illumina, San Diego, CA). The sequencing data were generated as fastq files, as previously described [19]. Briefly, the sequences were filtered for quality (sequences with >10 consecutive nucleotides with *Q* < 20 were eliminated) and mapped to the revised Cambridge Reference Sequence (rCRS), accession NC_012920. Identical reads were identified as being clonal and were considered only once, irrespective of the number of copies, toward variant calling. A variant call was made only if at least three non-clonal reads were carrying the variant, and minimum coverage of 10 was required at the variant (see Supplementary Figure S1 for coverage plots). Variants occurring on reads on one strand (with a skew greater than 0.1 or 10%) of the mtDNA were excluded to reduce errors further. The error rates in NextSeq reads are usually <1 in 1000 (phred score *Q* > 30). As the DNA bases are scored on a logarithmic scale, a phred score of 30 means a 1 in 1000 chance of errors, a phred score of 20 is a 1 in 100 chance of errors, etc. Therefore, if two independent reads line up with the same variant, then the chance of it being due to two coincident errors is approximately 1 in a million. Requiring at least three non-clonal reads reduces the error rate to well under one in a million. Nuclear contamination was estimated using sequences that map to repeat elements such as long interspersed nuclear elements (LINEs) and short interspersed nuclear elements (SINEs), which only occur in nDNA. This enables reliable estimation of the level of nDNA contamination. The fastq files have been uploaded to the Sequence Read Archive (SRA), BioProject accession number: PRJNA813947.

Concerning any residual nDNA contamination, the majority of the reads obtained were mitochondrial in origin; even if 50% of the reads are of nuclear origin, the NUclear MiTochondrial sequences (NUMTs) are a small fraction of the data (since they are a tiny fraction < 0.01% of the nuclear genome). Because contamination from NUMTs is greatly reduced, mapping the reads to the mitochondrial genome is reliable and needs no further corrections. Although the C315CC variant was identified at 62% heteroplasmy in the tumor specimen of patient 2, it is not addressed in this study as well as other studies due to technical difficulties and erroneous variant calls associated with the region located at rCRS positions 302–315 (ACCCCCCCTCCCCC) [13,31,33].

### 2.4. Determination of Relative mtDNA Copy Number

The samples were initially subjected to BamHI digestion to estimate mtDNA copy number in tumor and peri-normal tissue DNA extracts. Restriction endonuclease digestions were performed according to the manufacturers’ recommendations (New England Biolabs Inc., Ipswich, MA, USA). Briefly, 1 μg of DNA extract was digested with five units of BamHI restriction enzyme at 37 °C for 3 h. However, the patient 1 A14260G SNP removes the BamHI cut site; therefore, we used PvuII and BamHI double digests to maintain the 2.2 kb 18S nDNA internal control fragment and to linearize mtDNA from patient 1. In these reactions, 1 μg of DNA extract was digested with 5 U of each of BamHI and PvuII at 37 °C for 3 h in 1× NEBuffer r3.1. The 1 μg DNA digests were loaded onto 1.0% agarose gels in 1× TAE buffer (40 mM Tris, 20 mM acetic acid, 1 mM EDTA) without ethidium bromide and electrophoresed at 1.1 V/cm for 16 hr. The DNA samples were subjected to in-gel fragmentation/depurination, in-gel denaturation, Southern blotting, dual nDNA/mtDNA digoxigenin (DIG)-labeled probe hybridization (nDNA/RNA18SP4 probe nucleotide positions 101 to 600; mtDNA-specific probe, GenBank ID MK175431.1 nucleotide positions 168 to 606), membrane imaging, and band quantification as previously described [32,34].

### 2.5. Characterization of mtDNA Topoisomers

Using tumor and peri-normal tissue DNA extracts, mtDNA topoisomers were resolved via one-dimensional (1D) agarose gel electrophoresis (AGE), or 1D-AGE. An amount of 1 μg of each sample was digested with 5 U of BglII restriction enzyme at 37 °C for 3 h to fragment nDNA but not mtDNA. Each 1 μg sample was loaded into a lane of a 0.4% agarose gel made in 0.5× TBE (44.6 mM Tris, 44.5 mM boric acid, and 1 mM EDTA). The 1D gel was run for 16 h at 1.85 V/cm. After electrophoresis, the gel was stained in a 0.3 μg/mL ethidium bromide (EtBr) bath for 30 min. Next, in-gel denaturation of tumor and peri-normal tissue DNA extracts, Southern blotting, and hybridizations with the double-stranded DIG-labeled mtDNA-specific probe were conducted as previously described [34].

## 3. Results

Normal tissue mtDNA variants could arise via maternal inheritance or sporadic mutation. Here, we assume a normal tissue mtDNA variant is inherited and thus derived from the germline if found in both normal and tumor tissue samples. However, suppose a patient harbors a variant in both normal and tumor tissues not present in her haplogroup (the collection of mtDNA alleles inherited together). In that case, she could have somatically acquired the variant. Therefore, somatic cancer mutations were scored as variants present in tumors but absent in matched peri-normal samples.

### 3.1. mtDNA Mutations and Heteroplasmy Identified in Patient 1

Compared to the revised Cambridge Reference Sequence (rCRS), 17 mtDNA transition variants are shared between peri-normal and tumor tissues obtained from patient 1 (Supplementary Tables S1 and S2). Additionally, a silent C5375T heteroplasmic transition substitution was identified only in the peri-normal sample at 17% heteroplasmy, Supplementary Table S1. Utilizing the MITOMASTER mtDNA sequence analysis tool, the peri-normal and tumor mtDNA genomes from patient 1 are predicted to lie within the haplogroup branch V1a1; however, the peri-normal C5375T variant is not present in the haplogroup branch dataset [35]. The 17 shared variants include 9 A:T > G:C, 1 G:C > A:T, 3 T:A > C:G, and 4 C:G > T:A substitutions. Sixteen of the seventeen shared substitutions localize to haplogroup branch V1a1 with variable frequencies, while the remaining shared heteroplasmic C13662T is absent from the haplogroup. C13662T is listed as a synonymous single-nucleotide variant (SNV) in the NCBI Single-Nucleotide Polymorphism database, dbSNP. The CAROL (Combined Annotation scoRing toOL), APOGEE (pAthogenicity Prediction thrOugh loGistic modEl trEe), Mitochondrial tRNA Informatics Predictor (MitoTIP), and MutationAssessor in silico tools were utilized to predict whether a substitution could be pathogenic [36,37,38,39]. Of the 16 shared mtDNA variants localizing to V1a1, 8 are previously identified common changes from the rCRS [40], 4 are predicted to be neutral, 2 are synonymous substitutions, and 2 are SNVs. We suspect the 16 shared V1a1 substitutions are germline variants passed down from the patient’s mother. However, as the C13662T variant is absent from the haplogroup and is present in heteroplasmy in both normal and tumor tissues, it is possible this substitution was not inherited. In the normal tissue specimen, C13662T and C5375T were the only heteroplasmic substitutions identified; the remaining variants were nearly homoplasmic, ≥93% (100% is equivalent to homoplasmy).

**Figure 1:**
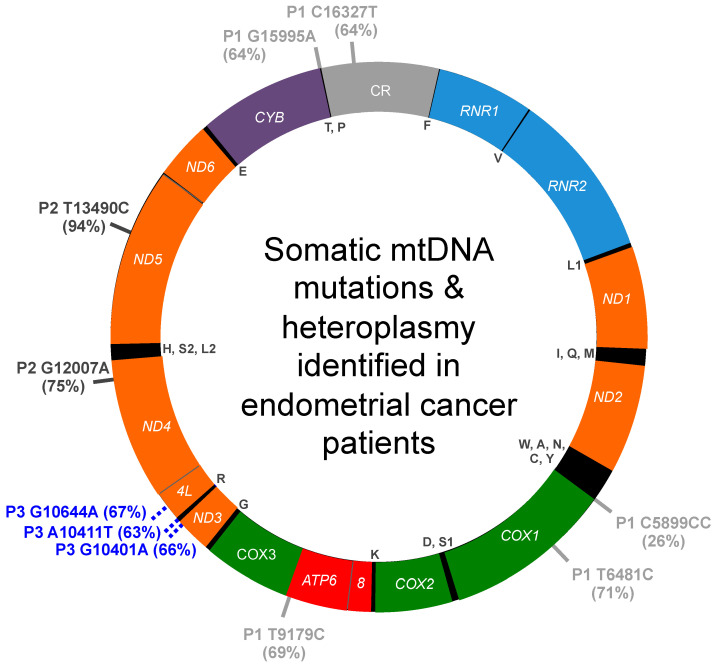
Summary of somatic mtDNA mutations and heteroplasmy identified in endometrial cancer patients. Variants for patients 1, 2, and 3 are indicated on the outside of the map by P1, P2, and P3, respectively, and the percentage heteroplasmy for each mutation is reported within brackets. The 13 genes encoding polypeptides of the mitochondrial OXPHOS machinery are highlighted in orange, OXPHOS complex I genes (NADH dehydrogenase, *ND1*, *ND2*, *ND3*, *ND4L*, *ND4*, *ND5*, and *ND6*); purple, OXPHOS complex III gene (cytochrome bc1 complex, *CYB*); green, OXPHOS complex IV genes (cytochrome c oxidase, *COX1*, *COX2*, and *COX3*); red, OXPHOS complex V genes (ATP synthase, *ATP6* and *ATP8*). The small 12S (*RNR1*) and large 16S (*RNR2*) ribosomal RNA genes are colored light blue, and the mtDNA control region (CR) is colored gray. The 22 tRNA genes are indicated by their respective single letter code of the amino acid residue inside the circular map.

Five heteroplasmic carcinosarcoma-specific mutations were identified in the tumor specimen obtained from patient 1, Table 1. These include one C5899CC insertion mutation and four transition mutations T6481C, T9179C, G15995A, and C16327T, Figure 1. Heteroplasmy for the 5 variants ranged from 26 to 71%. None of these substitutions are present in the V1a1 dataset; therefore, we predict they were somatically acquired during the malignant transformation process. In support of this idea, the four transition mutations have been reported associated with various cancer types including acute myeloid leukemia (LAML; T6481C), breast invasive carcinoma (BRCA; T6481C, G15995A), chronic lymphocytic leukemia (CLL; T9179C, C16327T), pancreatic adenocarcinoma (Panc-AdenoCA; T9179C, C16327T), prostatic adenocarcinoma (PRAD; C16327T) and stomach adenocarcinoma (STAD; C16327T) [31,33]. The T6481C/Val193Ala substitution alters an evolutionarily conserved amino acid residue in cytochrome c oxidase subunit 1 (COX1) that could affect mitochondrial function as predicted by MutationAssessor. Furthermore, T6481C is predicted to be deleterious and pathogenic by CAROL and APOGEE, respectively. C5899CC and C16327T localize to non-coding regions adjacent to the sites of mtDNA replication initiation. The T9179C substitution localizes to the ATP synthase *ATP6* gene and encodes a non-synonymous Val218Ala substitution that is predicted to be deleterious by CAROL and could affect mitochondrial function. Additionally, the G15995A variant localizes to the tRNA proline (*TRNP*) gene and is predicted to be pathogenic.

### 3.2. mtDNA Mutations and Heteroplasmy Identified in Patient 2

Using MITOMASTER, both the peri-normal and tumor mtDNA genomes derived from patient 2 are predicted to localize to haplogroup branch U5b2c2b (Supplementary Tables S3 and S4). Twenty-nine mtDNA transition variants that differ from the rCRS are shared between the patient’s peri-normal and tumor tissues. Additionally, a likely benign C960CC insertion variant in the *RNR1* gene, which is present in the haplogroup at 2.66%, is shared between the two tissues. The C960CC variant is heteroplasmic in both tissue types, peri-normal 53%, and tumor 68%. The remaining shared variants occurred at ≥90%. In addition, a silent peri-normal-specific homoplasmic A10262G *ND3* gene substitution that occurs in haplogroup branch U5b2c2b at 1.95% was also detected.

The 29 shared transition variants include 16 A:T > G:C, 3 G:C > A:T, 4 T:A > C:G, and 6 C:G > T:A substitutions. The transitions localize to the haplogroup branch, ranging from 1.33 to 99.69%. Many of these potential germline substitutions are associated with various human disorders. Four of the variants are associated with different cancers, C150T (cervical carcinoma risk [44]), A12308G (breast [45], renal, and prostate cancer risk [46]), C16192T (melanoma [47]), and C16270T (melanoma [47]). Additionally, A12308G is associated with chronic progressive external ophthalmoplegia (PEO) [48], stroke [49], cardiomyopathy [50], sporadic Creutzfeldt-Jakob disease (sCJD) [51], and altered brain pH [52]. Three variants are associated with sCJD (A11467G and G12372A [51]) and Leber hereditary optic neuropathy (LHON) (A13637G [53]). The remaining 22 transition substitutions include 4 non-synonymous variants (3 are commonly found in mtDNA genomes [40], and the fourth is predicted to be neutral), 8 that are silent/synonymous (3 are common), 3 SNVs found in the non-coding control region (CR; 2 are common), 6 that localize to the ribosomal RNA genes (3 are common), and 1 that is found in the mitochondrial tyrosine tRNA gene and is likely benign.

Two adenocarcinoma-specific transition mutations were identified in the tumor specimen obtained from patient 2, G12007A and T13490C, Table 2. The transition variants are absent from the U5b2c2b haplogroup branch dataset. G12007A is a synonymous variant previously reported associated with schizophrenia plus bipolar disorder and is present in the tumor sample at 75% heteroplasmy. T13490C is a 94% near-homoplasmy variant that changes an NADH dehydrogenase subunit 5 phenylalanine residue at position 385 to a serine, which is predicted to be damaging to the structure and function of the protein by MutationAssessor analysis. Additionally, T13490C is predicted to be deleterious and pathogenic by CAROL and APOGEE analyses. Therefore, we predict that these variants were somatically acquired during the malignant transformation process. In support of this hypothesis, both variants were recently reported in The Cancer Mitochondria Atlas (TCMA) associated with PRAD (G12007A), CLL (T13490C), and renal cell carcinoma (RCC, T13490C) [33].

### 3.3. mtDNA Mutations and Heteroplasmy Identified in Patient 3

Nine shared homoplasmic or near-homoplasmic mtDNA transition variants that deviate from the rCRS were identified in peri-normal and tumor tissue obtained from patient 3 (Supplementary Tables S5 and S6). The types of transitions identified include four A:T > G:C, two G:C > A:T, and three T:A > C:G. Four of the nine substitutions are commonly found in human mtDNA genomes [40]. Of the remaining five variants, three are found in the non-coding CR, one is in the *RNR1* gene, and one is a synonymous substitution in the *ND4* gene. The genomes derived from both tissues are predicted to localize to haplogroup branch H2a3a.

Three heteroplasmic mutations were identified in the adenocarcinoma specimen obtained from patient 3, including two transitions (G10401A, 66% heteroplasmy; G10644A, 67% heteroplasmy) and one transversion (A10411T, 63% heteroplasmy), Table 3. G10401A encodes a missense variant of the *ND3* gene that is predicted to be deleterious by CAROL but neutral by APOGEE analyses. The pathogenicity of the tRNA arginine A10411T and *ND4L* G10644A mutations are unclear as MitoTIP predicts A10411T is possibly benign, and conflicting interpretations of pathogenicity are reported for G10644A. However, as the three unique heteroplasmic mutations are absent from the H2a3a dataset, we predict these variants were somatically acquired during the cancer transformation process. In agreement with this notion, two of the mutations have been reported associated with other cancers such as thyroid carcinoma (THCA, G10401A), hepatocellular carcinoma (HCC, G10401A), and PRAD (G10644A) [31,33].

### 3.4. Tumor DNA Extracts from Patients 2 and 3 Harbor Altered mtDNA Copy Number

**Figure 2:**
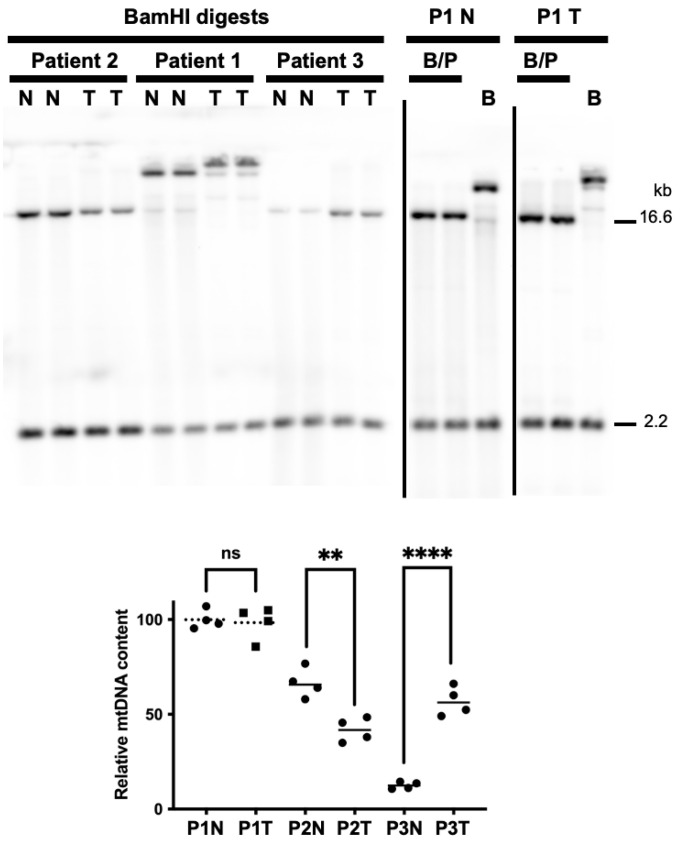
mtDNA copy number is altered in patient 2 and 3 tumors relative to peri-normal controls. BamHI (B) or BamHI and PvuII (B/P) digested tissue-extracted DNA samples were analyzed using Southern blotting and non-radioactive probe hybridization. The blots were simultaneously probed with the digoxigenin (DIG)-labeled 18S nDNA probe (2.2 kb band) and the mtDNA-specific probe (16.6 kb band). As previously described, bands were quantitated using the open-source image-processing package Fiji [32,34]. As the patient 1 A14260G SNP removes the BamHI cut site, PvuII and BamHI double digests were used to cut both the 18S and mtDNA loci. On the left-hand side blot, patient 1 samples digested with only BamHI are shown alongside patient 2 and 3 samples, but as the mtDNA banding patterns are different from the other samples, they were not used to quantitate mtDNA content. As the BamHI/PvuII digested patient 1 peri-normal DNA extract contained the highest average mtDNA to nDNA values, this sample was set to 100%, and the others were normalized to it. N, peri-normal; T, tumor; P1, patient 1; P2, patient 2; P3, patient 3. Significant differences between normal and tumor mtDNA contents were determined using *t*-tests, n = 4 for each sample set (see the graph below the blots; a representative blot is shown for each patient with n = 2 lanes shown for each sample, N and T); ****, *p* < 0.0001; **, *p* < 0.01; ns, not significantly different.

According to the strand displacement model of mtDNA replication, replisomes containing the DNA polymerase γ (Polγ) synthesize both the nascent heavy (H) and light (L) strands continuously without the formation of Okazaki-fragment-like replication products [56]. The two mtDNA strands are named H and L based on the ability to separate them on denaturing cesium chloride gradients. The H-strand is richer in G+T content making it heavier on density centrifugation [57,58]. The origin of H-strand DNA replication (O_H_) is located in the non-coding CR, and the origin of L-strand replication (O_L_) is located ~11,000 base pairs downstream of O_H_ [56]. To determine if mtDNA copy number changed in EC tumors relative to peri-normal tissues, we used our previously published Southern blot and dual digoxigenin (DIG)-labeled mtDNA and nDNA probe approach [32,34]. As the patient 1 A14260G SNP removes the mtDNA BamHI cut site, PvuII and BamHI double digests were used to cut both the nDNA (18S) and mtDNA loci in the patient 1 tumor and peri-normal samples. Surprisingly, the patient 1 tumor did not display a change in mtDNA content; however, a 37% reduction in patient 2 and a ~5-fold increase in patient 3 tumor mtDNA content were observed, Figure 2.

### 3.5. Endometrial Cancer Tumors Have Striking Differences in mtDNA Topoisomer Molecules

**Figure 3:**
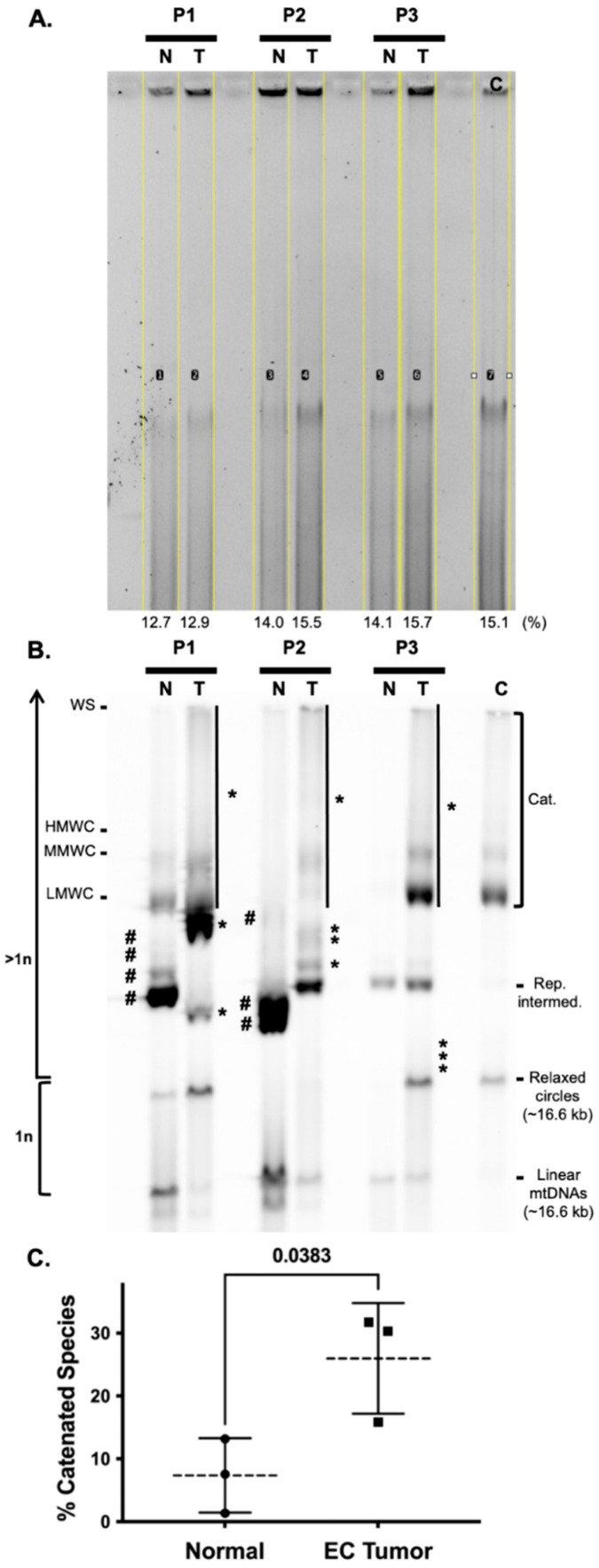
Endometrial cancer tumors have altered mtDNA topoisomers. Tissue-extracted DNA samples were digested with BglII to fragment nDNA (but not mtDNA). (**A**) One microgram of each DNA sample was loaded into each lane of a 0.4% TBE gel, and one-dimensional agarose gel electrophoresis (1D-AGE) was used to separate the various mtDNA topoisomers. Each lane of ethidium bromide-stained DNA signal was selected and quantitated as a percentage of the total size of all measured lanes using the open-source image-processing package Fiji, as previously described [32,34]. The percent values are reported below each lane. Note the average of all lanes is 14.3%, and the percent coefficient of variation (%CV) is 8.5% indicating that similar levels of DNA extract were loaded into each lane. (**B**) The blot was probed using the DIG-labeled mtDNA-specific probe. An SJCRH30 whole-cell DNA extract served as a control (C) to identify mtDNA topoisomers including catenanes (Cat.), the replication intermediate (Rep. intermed.), relaxed circles, and linear molecules. Note linear mtDNA and relaxed circles represent 1n mtDNA chromosomes, while species that migrate above the relaxed circles are >1n. Catenated species (Cat.) include mtDNA well species (WS), high-molecular-weight catenanes (HMWC), mid-range MW catenanes (MMWC), low MW catenanes (LMWC), and the smear of the signal down the lane from WS to LMWC. Catenanes are emphasized by the black vertical lines next to the tumor (T) extracted DNA samples for patients 1, 2, and 3, P1, P2, and P3, respectively. Topoisomer species seen in peri-normal tissue (N) but not in endometrial cancer (EC) tumors are emphasized with #, while those seen in T, but not N tissues are emphasized with *. (**C**)**.** The levels of catenated species were quantitated using the open-source image-processing package Fiji. Each lane of catenane signal on the blot was quantitated as a percentage of the total size of all measured lanes. The data are presented as the mean values ± errors as standard deviations (SDs). Mean, and SD values were calculated from the n = 3 sets of N and T DNA extracts from P1, P2, and P3. The difference between normal and tumor tissue catenane levels was determined using a student’s t-test. The *p*-value of 0.0383 is considered significant (*p* < 0.05).

Initially, we digested patient 1 DNA samples with only BamHI for the mtDNA copy number analysis. In these experiments, we noticed that the mtDNA remained undigested and that various topological isoforms or topoisomers were present in the normal and tumor samples. However, an increase in high-molecular-weight (HMW) species was observed in the tumor sample, Figure 2. Elegant studies have demonstrated that among different cell types and tissues derived from humans and mice, there exist major mtDNA topoisomers (e.g., catenanes, relaxed circles, and linear molecules), but the molecules can be distributed differently, and additional structures can be seen depending on the cell type or tissue [59,60,61,62]. Based on the BamHI single digests of the patient 1 samples, we speculated that EC tumors harbor different distributions of topoisomers relative to peri-normal tissue due to altered mtDNA maintenance. The samples were subjected to one-dimensional 0.4% agarose gel electrophoresis (1D-AGE), Southern blotting, and non-radioactive probe hybridization to investigate mtDNA topoisomers. Strikingly, mtDNA topoisomers were altered in all EC tumor mtDNA samples relative to their matched peri-normal specimens, Figure 3.

In patients 1 and 2, four and three species seen in the peri-normal samples were absent in the tumors (see the bands emphasized with # in Figure 3). Additionally, all tumor specimens harbor ≥ 3 new species that were absent in the matched peri-normal samples (see the bands emphasized with *). On average, the patient tumor mtDNA catenated species were significantly increased by 3.5 fold relative to the peri-normal catenanes.

## 4. Discussion

Studies have demonstrated negative (purifying) selection acting on human mtDNA in the germline [63,64,65]. On the other hand, recent reports indicate that cancer cells harbor heteroplasmic mtDNA somatic mutations that encode pathogenic substitutions or protein truncations [13,14,31]. The accumulation of pathogenic somatic mtDNA mutations in cancer could result from a relaxed or positive selection. The caveat with either of these selections is that the proportion of mtDNA molecules harboring damaging mutations cannot increase to homoplasmy (or near homoplasmy) without harming cancer cell mitochondrial metabolism. One possibility is that the proportion of wild-type and mutant mtDNA molecules can be tuned to optimize cancer cell metabolism, growth, and proliferation.

In a normal cell, mtDNA heteroplasmic mutations cluster within the non-coding CR while tumor-specific somatic mutations tend to be evenly distributed across coding and non-coding regions [31]. Additionally, protein-altering variants in kidney chromophobe and thyroid carcinomas show strong evidence of positive selection acting on their mtDNA genomes [31]. Therefore, we hypothesize that the acquisition of stable heteroplasmic mtDNA mutations optimize mitochondrial metabolism and drive the progression of EC. To initially test this hypothesis, we sequenced mtDNA genomes from three matched peri-normal and tumor tissues using Mseek NGS to determine tumor-specific mutations and heteroplasmy levels. As a result, we identified somatic mtDNA heteroplasmic substitutions in all three tumor specimens.

The sample containing the most heteroplasmic mtDNA mutations was the carcinosarcoma tumor from patient 1. As mentioned earlier, four out of five of the substitutions have been previously associated with different forms of cancers. As judged by MutationAssessor, T6481C and T9179C encode missense variants predicted to alter the functions of the COX complex and the ATP synthase, respectively. Similarly, G15995A is predicted to be a pathogenic mutation that alters the secondary structure of the mitochondrial tRNA proline. Two of the patient 1 carcinosarcoma-specific mutations localized to non-coding regions near O_H_ and O_L_, C16327T and C5899CC, respectively. Although patient 1 mtDNA content was similar in the tumor and peri-normal tissues, in all the tumors mtDNA catenane levels were increased and tumor-specific topoisomers were revealed by 1D-AGE (Figure 2 and Figure 3). These findings again support the idea that the proportion of WT and mutant mtDNA may tune cancer cell metabolism. The mtDNA catenated network is suggested to be a replicating population or products of mtDNA replication [59,66]; however, total mtDNA content remained unchanged in the patient 1 tumor and was decreased in patient 2 (Figure 2). This suggests mtDNA homeostasis (synthesis by Polγ and degradation by the degradosome) might be altered in EC. Southern blotting served as a powerful tool to characterize and identify the human degradosome, the mitochondrial machinery that degrades mtDNA [67]. In the mtDNA degradosome model, the p140 catalytic subunit of Polγ harboring the 3′-5′ exonuclease activity, the 5′-3′ mtDNA Twinkle helicase, and the 5′-3′ exonuclease MGME1 work in concert to quickly degrade linear mtDNA molecules. Perhaps deregulation of the mtDNA replisome and degradosome contribute to the complexities of mtDNA copy number and topoisomers in EC. Finally, as the patient 1 T6481C, T9179C, G15995A, and C16327T mutations occur at similar heteroplasmy levels, we predict they exist on identical molecules. In contrast, C5899CC occurs at lower levels relative to the other mutations and likely exists on separate mtDNAs.

The remaining two tumor specimens were grade 1 and 3 adenocarcinomas obtained from patients 2 and 3. Each of these tumors harbors two mutations previously reported associated with different forms of cancer. The adenocarcinoma-specific mutations are heteroplasmic; however, 1 of the 2 variants from the grade 1 tumor is near homoplasmy and predicted to be damaging to the function of the NADH dehydrogenase, T13490C/Phe385Ser. The other *ND4* G12007A grade 1 tumor variant codes for a synonymous substitution. Interestingly, the G10644A, A10411T, and G10401A adenocarcinoma-specific variants detected in the grade 3 tumor cluster within a region of <245 bp and occur at similar levels of heteroplasmy, suggesting that they localize together on the mtDNA genome. The G10401A mutation is predicted to be deleterious by CAROL analysis.

On average, we detected 3.3 mutations per EC tumor, which agrees well with the three mutations per EC tumor reported for the 51 uterus adenocarcinoma samples in The Cancer Mitochondria Atlas (TCMA) [33]. TCMA surveyed 2536 high-quality matched cancer and control sample pairs from the Pan-Cancer Analysis of Whole Genomes Consortium covering 38 specific cancer types and identified 7611 somatic mtDNA substitutions and 930 small indels. Of the 7611 variants identified, >85% were heteroplasmic. Additionally, in contrast with nDNA mutations where cancer type-specific signatures are seen, mtDNA mutations are similar across different tumor types, and most of the mutations display strand bias with predominantly G > A and T > C substitutions on the L-strand [33]. Interestingly, this strand bias occurs despite the relative depletion of guanines and thymines on the L-strand. In agreement with TCMA findings, we detected a total of nine heteroplasmic variants plus one near homoplasmic T13490C variant in the three EC tumor specimens. Additionally, in alignment with a previous report on somatic mtDNA mutations in human cancer [13], eight transition mutations constituted the bulk of the somatic mutational signatures in the 3 EC tumors, 80% of the mtDNA tumor variants. Indeed, out of the ten EC mutations detected in the three tumors, three are T > C (L-strand changes in two of the tumors), and four are G > A transitions (L-strand changes found in all three tumors). The remaining transition was a C > T change on the L-strand. These transitions likely arose from erroneous incorporation by the replicative Polγ or deamination events during strand-displacement mtDNA replication as previously described [13,68,69]. The remaining mutations consisted of an insertion and a transversion. We predict Polγ also created these two mutations during mtDNA genome replication. The C5899CC insertion occurs at the 3′-end of a homopolymer of 5 Cs that may have caused Polγ slippage during DNA synthesis. As mentioned above, the A10411T transversion clusters with two other mutations within a region of <245 bp and perhaps resulted from a burst of uncorrected Polγ misincorporation events.

In addition to three CR SNVs (Supplementary Table S1), patient 1 harbors two tumor-specific mutations that localize to non-coding regions, C16327T and C5899CC. The C16327T substitution is located downstream of O_H_ in the non-coding 7S DNA region and could impact leading strand mtDNA replication initiation. One hypothesis is that 7S DNA can be utilized as a primer to initiate H-strand replication [70]. Perhaps the C16327T 7S DNA sequence change was selected for in a founding carcinosarcoma cell to modify mtDNA replication initiation and, by extension, mitochondrial metabolism, and cancer progression. As mentioned above, this variant is associated with STAD, CLL, PRAD, and Panc-AdenoCA. The C5899CC insertion is located downstream of O_L_. Possibly, C5899CC was subjected to a similar selection as C16327T. Furthermore, in addition to three CR SNVs, patient 2 has three disease-associated CR variants, C150T (cervical carcinoma, HPV infection risk), C16192T (melanoma), and C16270T (melanoma) (Supplementary Table S3). Additionally, four CR SNVs were identified in patient 3 (Supplementary Table S5). Variants near the mtDNA origins of replication could alter mtDNA maintenance, and elevated mtDNA content has been demonstrated to be associated with an increased risk of lymphoma [71].

To make further comparisons with additional samples, we tabulated uterine tumor data from the Ju et al. study that investigated somatic mtDNA mutations from a collection of 31 tumor types [13]. Using a cutoff of at least 100 reads in the matched uterine normal and tumor tissue pairs, we consider ten additional sets from Ju et al., Table 4. Percent heteroplasmy was calculated as (variant read count/(variant read count + WT read count)) × 100. In agreement with our results, and the concept of erroneous incorporation by the replicative Polγ, sixteen L-strand transition mutations were identified in the ten tumors, 5 T > C, 10 G > A, and 1 C > T. Nine out of the sixteen mutations have been found associated with cancers other than uterine carcinoma. Ten of the sixteen mutations (~63%) are found at ≤92% heteroplasmy (from 12 to 92%), and eight of the ten sample indexes (tumors) harbor at least one of these substitutions. Sample indexes 5157 and 5167 harbor variants closer to homoplasmy at ≥94%. Pooling the Table 4 data with our data for a total of thirteen tumors than on average, there are two mutations per EC tumor, and 19/26 (73%) of the variants are heteroplasmic (≤92% variant). These findings are similar to the TCMA finding of three mutations per uterine adenocarcinoma tumor, with >85% of the variants being heteroplasmic. Furthermore, in Table 4, five of the variants encode missense mutations (two are predicted to be pathogenic), three alter a tRNA (one is confirmed pathogenic, and two are predicted to be possibly pathogenic), and one is a nonsense mutation (G13417A, changing glycine/GGA to stop/AGA resulting in a truncated 360 amino acid residue ND5 protein rather than the wild-type 603 amino acid protein). Although only one nonsense mutation was found in this analysis of uterine tumors, the mutation’s localization to the *ND5* gene agrees with previous work demonstrating that tumor mtDNA truncating mutations preferentially impact complex I [72]. Of the remaining seven variants in Table 4, three are synonymous substitutions, two are in the non-coding control region, one is in the 16S rRNA gene (*RNR2*), and another is in the non-coding O_L_ region. Again, pooling the Table 4 data with the results of this study gives 10 missense variants, 5 tRNA variants, 5 non-coding region variants, 1 nonsense variant, 1 *RNR2* variant, and 4 silent variants. Of the ten pooled missense variants, seven are associated with genes encoding subunits of complex I, two with complex V, and one with complex IV.

Future studies could investigate the effect of predicted heteroplasmic pathogenic tRNA and missense mutations by generating cybrids and measuring outputs such as cell proliferation, bioenergetics, apoptosis, and tumorigenicity in xenograft tumor models. While our sample size of three EC tumors is small, our data agree with other published studies demonstrating that somatic mtDNA heteroplasmy is a unique feature of cancer cell genomes. Importantly, our results suggest that increased levels of mtDNA catenanes may serve as a useful diagnostic tool for EC. Future work will explore mtDNA topoisomers in an expanded set of EC tumor and peri-normal tissues. Six to thirteen percent of all ECs are reported to recur, and the prognosis for recurrent disease is poor, with median survival barely exceeding one year [73,74]. Currently, there is no EC biomarker in routine use [75]. A biomarker would be a useful diagnostic method to complement methods used to diagnose EC and recurrent EC, such as transvaginal ultrasound and endometrial biopsy. Furthermore, recent evidence suggests that cell-free mtDNA in the bloodstream has prognostic value in several human diseases, including cancer [76,77]. Perhaps EC mtDNA could be detected in blood serum, which contains a relatively low amount of total DNA? Additionally, in comparison to nDNA, mtDNA is circular (resistant to exonuclease degradation), short in length (16.6 kb), and present at high copy number (polyploidy), which are desirable features for a NGS biomarker. Diagnosing recurrent cancer as early as possible would have substantial clinical implications. Patients with type II cancers could have their blood tested at regular intervals for mtDNA heteroplasmy as part of a monitoring program. Therefore, we propose that mtDNA heteroplasmy may serve as a valuable biomarker of EC. As we detected mtDNA heteroplasmy in grade 1 and 3 tumors, with further evidence, heteroplasmic mutations might prove useful as cancer biomarkers and assist clinicians in the determination of a patient’s prognosis and personalized treatment plan.

## Figures and Tables

**Table 1:** Patient 1 unique tumor mtDNA somatic variants and heteroplasmy levels.

MtDNA nt change ^a^	Location ^b^	Coverage ^c^	%Variant ^d^	Remarks ^e^
C5899CC	NC5, adjacent O_L_ and *TRNY*	714	26	Insertion of C, potential effect on DNA rep. initiation?
T6481C	*COX1*/V193A	794	71	Missense variant, deleterious (1.0) ^f^; pathogenic (0.56) ^g^; functional impact medium (2.34) ^h^; associated with LAML, BRCA/Breast AdenoCA [31,33]
T9179C	*ATP6*/V218A	767	69	Missense variant, deleterious (0.99) ^f^; neutral (0.4) ^g^; functional impact medium (2.91) ^h^; associated with CLL, Panc-AdenoCA [33]
G15995A	*TRNP*	756	64	Likely pathogenic (MitoTIP80%) ^i^, mitochondrial cytopathy [41]; associated with BRCA/Breast-AdenoCA [31,33]
C16327T	ATT, CR:HVS1,7S	489	64	Potential effect on DNA rep. initiation? associated with STAD, CLL, PRAD, Panc-AdenoCA [31,33]

^a^ Nucleotide (nt) positions are numbered according to the revised Cambridge Reference Sequence (rCRS) light-strand, NC_012920.1. rCRS nucleotides are listed on the left while variants are listed on the right. ^b^ Light-strand origin of mtDNA replication (O_L_); non-coding region 5 (NC5); tRNA tyrosine (*TRNY*); tRNA proline (*TRNP*); The membrane attachment site (ATT) has overlap with the non-coding control region (CR) containing the 7S DNA (7S), and the hypervariable segment 1 (HVS1). See https://www.mitomap.org/foswiki/bin/view/MITOMAP/GenomeLoci (accessed on 1 December 2019). Other gene names are explained in the legend for Figure 1. ^c^ NGS coverage at the indicated position. ^d^ The percentage of the variant in the plus strand reads, heteroplasmy; 100% represents homoplasmy. ^e^ Predictions regarding mutation pathogenicity were done using CAROL (Combined Annotation scoRing toOL), APOGEE (pAthogenicity Prediction thrOugh loGistic modEl trEe), MutationAssessor, or Mitochondrial tRNA Informatics Predictor (MitoTIP). DNA replication (DNA rep.). ^f^ The CAROL score combines information from the bioinformatics tools PolyPhen-2 [42] and SIFT [43] to predict the effect of non-synonymous coding variants (https://www.sanger.ac.uk/tool/carol/; accessed on 1 March 2022). The CAROL scores range between 0 and 1, with scores > 0.98 considered deleterious. ^g^ The APOGEE score was determined using the MitImpact 3D bioinformatics resource at https://mitimpact.css-mendel.it/ (accessed on 1 March 2022) [36,37]. APOGEE is a Logistic Model Tree (LMT)-based consensus classifier, a machine learning technique that consists of a combination of decision trees and logistic regressions at the leaves. APOGEE handles neutral and pathogenic pathogenicity classes. Mutations are considered as instances of several different predictors (e.g., PolyPhen2, MutationAssessor, and PROVEAN). Once the class is defined, a bootstrap strategy is implemented that randomly selects 70% of the pathogenic mutations, and the same number of neutral mutations are considered. For 100 iterations, the algorithm is run to sample the training set, estimate the LMT, and predict the pathogenicity of the mutations stored in the dataset. An estimate of the variant’s pathogenicity is given during each iteration and values are summarized by calculating a probability mean. A variant is deemed harmful if the mean of being harmful on 100 runs is >0.5. ^h^ MutationAssessor predicts the potentially deleterious impact of a DNA mutation that changes a protein’s amino acid residue. To make this prediction, multiple sequence alignments of homologous proteins are grouped into families and subfamilies. Using the conservation pattern information generated from the alignments, MutationAssessor generates a functional impact (FI) score to rate a mutation as either having a predicted functional impact on the encoded protein (high or medium) or not (low or neutral, non-functional substitution). FI scores are listed within brackets in the table. ^i^ MitoTIP scoring, an in silico tool embedded into Mitomaster for predicting pathogenicity of novel mitochondrial tRNA variants [38]. Each possible nt change was scored, and the scores have been interpreted within quartiles: (1) likely pathogenic (>75–100%), (2) possibly pathogenic (>50–75%), (3) possibly benign (>25–50%), and (4) likely benign (0–25%).

**Table 2:** Patient 2 unique tumor mtDNA somatic variants and heteroplasmy levels.

MtDNA nt Change ^a^	Location	Coverage	%Variant	Remarks
G12007A	*ND4*/W416W	424	75	Synonymous variant; significantly associated with SZ + BD ^b^ [54]; associated with PRAD [33]
T13490C	*ND5*/F385S	516	94	Missense variant, deleterious (1.0) ^c^; pathogenic (0.63) ^d^; Functional impact medium (3.425) ^e^; associated with CLL, RCC [33]

^a^ Row headings are as defined in the footnote for Table 1 and gene names are explained in the Figure 1 legend. ^b^ Schizophrenia (SZ) + bipolar disorder (BD). ^c^ The CAROL and ^d^ APOGEE scores were determined as described in Table 1 footnotes. ^e^ MutationAssessor predicts the potentially deleterious impact of a DNA mutation that changes a protein’s amino acid residue (see Table 1 footnote).

**Table 3:** Patient 3 unique tumor mtDNA somatic variants and heteroplasmy levels.

MtDNA nt Change ^a^	Location ^b^	Coverage	%Variant	Remarks
G10401A	*ND3*/E115K	556	66	Missense variant, deleterious (0.99) ^c^; neutral (0.31) ^d^; associated with THCA, HCC, Ovary-AdenoCA [31,33]
A10411T	*TRNR*	540	63	Assoc. w/cardiomyopathy [55]; possibly benign(MitoTIP26.4%) ^e^
G10644A	*ND4L*/V59M	658	67	Missense variant, neutral (0.45) ^c^; neutral (0.3) ^d^; conflicting interpretations of pathogenicity (NC_012920.1(MT-ND4L):m.10644G>A) ^f^; associated with PRDA [33]

^a^ Row headings are as defined in the footnote for Table 1. ^b^ tRNA arginine (*TRNR*); other gene names and abbreviations are explained in the legend for Figure 1 and Table 1 footnote. ^c^ The CAROL and ^d^ APOGEE scores were determined as described in Table 1 footnotes. ^e^ MitoTIP scoring (see Table 1 footnote for explanation). ^f^ Conflicting interpretations of pathogenicity listed in ClinVar.

**Table 4:** mtDNA heteroplasmy identified in uterine tumors from Ju et al. 2014 [13]. Only tumor tissue samples that had read counts >100 (variant or WT) and matched normal tissue WT read counts > 100 were analyzed. (The matched normal levels of mtDNA heteroplasmy were all less than 0.4%.)

Sample Index (See Supp. File 2 in [13])	MtDNA nt Change ^a^	Location ^b^	%Variant	Remarks ^c^
5128	T5506C	*ND2*/I346T	91	Missense, neutral (0.77) ^d^; neutral (0.45) ^e^; Uterus-AdenoCA [33]
5139	T11875C	*ND4*/T372T	92	Synonymous, UCEC, Eso-AdenoCA [31,33]
5139	G13531A	*ND5*/A399T	93	Missense, neutral (0.52) ^d^; neutral (0.29) ^e^; UCEC, Panc-Endorine, Soft Tissue-Liposarc, HCC [31,33]
5150	G4308A	*TRNI*	46	Confirmed pathogenic (MitoTIP82.1%) ^f^, UCEC, Breast AdenoCA, Kidney-ChRCC [31,33]
5155	G14410A	*ND6*/V88V	35	Synonymous, UCEC [31]
5157	C14112T	*ND5*/F592F	96	Synonymous, UCEC/Uterus-AdenoCA [31,33]
5160	T152C	ATT, CR: HVS2, 7S, O_H_	88	LGG, LUAD/ Lung-AdenoCA, SARC, SKCM, THCA, BLCA/ Bladder-TCC, BRCA/Breast-AdenoCA, ESCA, PRAD/Prost-AdenoCA, STAD, UCEC, Lymph-CLL, Panc-AdenoCA, Soft Tissue-Liposarc, skin melanoma [31,33]
5160	G1642A	*TRNV*	80	Possibly pathogenic (MitoTIP74.3%) ^f^, HNSC, UCEC, Head-SCC, Panc-AdenoCA, Thy-AdenoCA, HCC [31,33]
5160	G13417A	*ND5*/G361TERM	51	Nonsense mutation, LUAD, UCEC, HNSC [31]
5161	G5560A	*TRNW*	12	Possibly pathogenic (MitoTIP62.3%) ^f^, THCA/Thy-AdenoCA, Kidney-ChRCC, Ovary-AdenoCA [31,33]
5163	T9062C	*ATP6*/L179P	14	Missense, neutral (0.92) ^d^; neutral (0.25) ^e^; UCEC/Uterus-AdenoCA [31,33]
5165	G529A	CR: HVS3, TFH	99	
5165	G2553A	*RNR2*	17	UCEC, Panc-AdenoCA, Prost-AdenoCA [31,33]
5165	G5753A	O_L_	98	HCC, Uterus-AdenoCA [33]
5165	G14453A	*ND6*/A74V	98	Missense, deleterious (1.0) ^d^; pathogenic (0.81) ^e^; Kidney-ChRCC, Ovary-AdenoCA, CNS-Medullo, HCC, Biliary-AdenoCA [33]
5167	T14325C	*ND6*/N117D	94	Missense, neutral (0.56) ^d^; pathogenic (0.54) ^e^; UCEC [31]

^a^ Row headings are as defined in the footnote for Table 1. ^b^ tRNA isoleucine (*TRNI*); tRNA valine (*TRNV*); tRNA tryptophan (*TRNW*); TFH, mtTF1/TFAM binding site; other gene names and abbreviations are explained in the legend for Figure 1 and Table 1 footnote. ^c^ UCEC, uterine corpus endometrial carcinoma; AdenoCA, adenocarcinoma; Eso, esophageal; Panc, pancreas; Liposarc, liposarcoma; ChRCC, chromophobe renal cell carcinoma; LGG, brain lower-grade glioma; LUAD, lung adenocarcinoma; SARC, Sarcoma; SKCM, skin cutaneous melanoma; THCA, thyroid carcinoma; Thy, thyroid; BLCA, bladder urothelial carcinoma; TCC, transitional cell carcinoma; SCC, squamous cell carcinoma; ESCA, esophageal carcinoma; PRAD, prostate adenocarcinoma; Prost, prostate; HNSC, head and neck squamous cell carcinoma; CNS-Medullo, central nervous system medulloblastoma. ^d^ The CAROL and ^e^ APOGEE scores were determined as described in the Table 1 footnotes. ^f^ MitoTIP scoring (see Table 1 footnote for explanation).

## Data Availability

The fastq files will be submitted to the NCBI Sequence Read Archive (SRA).

## Supplementary Materials

The following supporting information can be downloaded at: https://www.mdpi.com/article/10.3390/life12040562/s1, Figure S1: Mseek deep sequencing read depth; Table S1: Patient 1 peri-normal tissue mtDNA changes and heteroplasmy levels, haplogroup branch V1a1; Table S2: Patient 1 tumor tissue mtDNA changes and heteroplasmy levels, haplogroup branch V1a1; Table S3: Patient 2 peri-normal tissue mtDNA changes and heteroplasmy levels, haplogroup branch U5b2c2b; Table S4: Patient 2 tumor tissue mtDNA changes and heteroplasmy levels, haplogroup branch U5b2c2b; Table S5: Patient 3 peri-normal mtDNA changes and heteroplasmy levels, haplogroup branch H2a3a; Table S6: Patient 3 tumor mtDNA changes and heteroplasmy levels, haplogroup branch H2a3a.

## References

[1] Braun M.M., Overbeek-Wager E.A., Grumbo R.J. (2016). Diagnosis and Management of Endometrial Cancer. Am. Fam. Physician.

[2] Zhang S., Gong T.T., Liu F.H., Jiang Y.T., Sun H., Ma X.X., Zhao Y.H., Wu Q.J. (2019). Global, Regional, and National Burden of Endometrial Cancer, 1990-2017: Results From the Global Burden of Disease Study, 2017. Front. Oncol..

[3] Sternberg A.K., Buck V.U., Classen-Linke I., Leube R.E. (2021). How Mechanical Forces Change the Human Endometrium during the Menstrual Cycle in Preparation for Embryo Implantation. Cells.

[4] Stewart B.W., Wild C. (2014). International Agency for Research on Cancer, World Health Organization. World Cancer Report 2014.

[5] Zahnd W.E., Hyon K.S., Diaz-Sylvester P., Izadi S.R., Colditz G.A., Brard L. (2018). Rural-urban differences in surgical treatment, regional lymph node examination, and survival in endometrial cancer patients. Cancer Causes Control..

[6] Warburg O. (1956). On the origin of cancer cells. Science.

[7] Liberti M.V., Locasale J.W. (2016). The Warburg Effect: How Does it Benefit Cancer Cells?. Trends Biochem. Sci..

[8] Weinberg S.E., Chandel N.S. (2015). Targeting mitochondria metabolism for cancer therapy. Nat. Chem. Biol..

[9] Guerra F., Kurelac I., Cormio A., Zuntini R., Amato L.B., Ceccarelli C., Santini D., Cormio G., Fracasso F., Selvaggi L. (2011). Placing mitochondrial DNA mutations within the progression model of type I endometrial carcinoma. Hum. Mol. Genet.

[10] Musicco C., Cormio G., Pesce V., Loizzi V., Cicinelli E., Resta L., Ranieri G., Cormio A. (2018). Mitochondrial Dysfunctions in Type I Endometrial Carcinoma: Exploring Their Role in Oncogenesis and Tumor Progression. Int. J. Mol. Sci..

[11] Chang M. (2011). Dual roles of estrogen metabolism in mammary carcinogenesis. BMB Rep..

[12] Gammage P.A., Frezza C. (2019). Mitochondrial DNA: The overlooked oncogenome?. BMC Biol..

[13] Ju Y.S., Alexandrov L.B., Gerstung M., Martincorena I., Nik-Zainal S., Ramakrishna M., Davies H.R., Papaemmanuil E., Gundem G., Shlien A. (2014). Origins and functional consequences of somatic mitochondrial DNA mutations in human cancer. Elife.

[14] Stewart J.B., Alaei-Mahabadi B., Sabarinathan R., Samuelsson T., Gorodkin J., Gustafsson C.M., Larsson E. (2015). Simultaneous DNA and RNA Mapping of Somatic Mitochondrial Mutations across Diverse Human Cancers. PLoS Genet.

[15] Schon E.A., DiMauro S., Hirano M. (2012). Human mitochondrial DNA: Roles of inherited and somatic mutations. Nat. Rev. Genet.

[16] Santos J.H., Hunakova L., Chen Y., Bortner C., Van Houten B. (2003). Cell sorting experiments link persistent mitochondrial DNA damage with loss of mitochondrial membrane potential and apoptotic cell death. J. Biol. Chem..

[17] Young M.J., Copeland W.C. (2016). Human mitochondrial DNA replication machinery and disease. Curr. Opin. Genet Dev..

[18] Young M.J., Humble M.M., DeBalsi K.L., Sun K.Y., Copeland W.C. (2015). POLG2 disease variants: Analyses reveal a dominant negative heterodimer, altered mitochondrial localization and impaired respiratory capacity. Hum. Mol. Genet.

[19] Jayaprakash A.D., Benson E.K., Gone S., Liang R., Shim J., Lambertini L., Toloue M.M., Wigler M., Aaronson S.A., Sachidanandam R. (2015). Stable heteroplasmy at the single-cell level is facilitated by intercellular exchange of mtDNA. Nucleic Acids Res..

[20] He Y., Wu J., Dressman D.C., Iacobuzio-Donahue C., Markowitz S.D., Velculescu V.E., Diaz L.A., Kinzler K.W., Vogelstein B., Papadopoulos N. (2010). Heteroplasmic mitochondrial DNA mutations in normal and tumour cells. Nature.

[21] Bayona-Bafaluy M.P., Manfredi G., Moraes C.T. (2003). A chemical enucleation method for the transfer of mitochondrial DNA to rho(o) cells. Nucleic Acids Res..

[22] King M.P., Attardi G. (1996). Isolation of human cell lines lacking mitochondrial DNA. Methods Enzymol..

[23] Carelli V., Vergani L., Bernazzi B., Zampieron C., Bucchi L., Valentino M., Rengo C., Torroni A., Martinuzzi A. (2002). Respiratory function in cybrid cell lines carrying European mtDNA haplogroups: Implications for Leber’s hereditary optic neuropathy. Biochim. Biophys. Acta.

[24] Heller S., Schubert S., Krehan M., Schafer I., Seibel M., Latorre D., Villani G., Seibel P. (2013). Efficient repopulation of genetically derived rho zero cells with exogenous mitochondria. PLoS ONE.

[25] Kwong J.Q., Henning M.S., Starkov A.A., Manfredi G. (2007). The mitochondrial respiratory chain is a modulator of apoptosis. J. Cell Biol..

[26] Ishikawa K., Takenaga K., Akimoto M., Koshikawa N., Yamaguchi A., Imanishi H., Nakada K., Honma Y., Hayashi J. (2008). ROS-generating mitochondrial DNA mutations can regulate tumor cell metastasis. Science.

[27] Seyfried T.N., Flores R.E., Poff A.M., D’Agostino D.P. (2014). Cancer as a metabolic disease: Implications for novel therapeutics. Carcinogenesis.

[28] Gaude E., Schmidt C., Gammage P.A., Dugourd A., Blacker T., Chew S.P., Saez-Rodriguez J., O’Neill J.S., Szabadkai G., Minczuk M. (2018). NADH Shuttling Couples Cytosolic Reductive Carboxylation of Glutamine with Glycolysis in Cells with Mitochondrial Dysfunction. Mol. Cell.

[29] Young M.J., Jayaprakash A.D., Young C.K.J. (2019). Analysis of Mitochondrial DNA Polymorphisms in the Human Cell Lines HepaRG and SJCRH30. Int. J. Mol. Sci..

[30] Wallace D.C., Stugard C., Murdock D., Schurr T., Brown M.D. (1997). Ancient mtDNA sequences in the human nuclear genome: A potential source of errors in identifying pathogenic mutations. Proc. Natl. Acad. Sci. USA.

[31] Grandhi S., Bosworth C., Maddox W., Sensiba C., Akhavanfard S., Ni Y., LaFramboise T. (2017). Heteroplasmic shifts in tumor mitochondrial genomes reveal tissue-specific signals of relaxed and positive selection. Hum. Mol. Genet.

[32] Young C.K.J., Wheeler J.H., Rahman M.M., Young M.J. (2021). The antiretroviral 2′,3′-dideoxycytidine causes mitochondrial dysfunction in proliferating and differentiated HepaRG human cell cultures. J. Biol. Chem..

[33] Yuan Y., Ju Y.S., Kim Y., Li J., Wang Y., Yoon C.J., Yang Y., Martincorena I., Creighton C.J., Weinstein J.N. (2020). Comprehensive molecular characterization of mitochondrial genomes in human cancers. Nat. Genet..

[34] Wheeler J.H., Young C.K.J., Young M.J. (2019). Analysis of Human Mitochondrial DNA Content by Southern Blotting and Nonradioactive Probe Hybridization. Curr. Protoc. Toxicol..

[35] Lott M.T., Leipzig J.N., Derbeneva O., Xie H.M., Chalkia D., Sarmady M., Procaccio V., Wallace D.C. (2013). mtDNA Variation and Analysis Using Mitomap and Mitomaster. Curr. Protoc. Bioinform..

[36] Castellana S., Fusilli C., Mazzoccoli G., Biagini T., Capocefalo D., Carella M., Vescovi A.L., Mazza T. (2017). High-confidence assessment of functional impact of human mitochondrial non-synonymous genome variations by APOGEE. PLoS Comput. Biol..

[37] Castellana S., Biagini T., Petrizzelli F., Parca L., Panzironi N., Caputo V., Vescovi A.L., Carella M., Mazza T. (2021). MitImpact 3: Modeling the residue interaction network of the Respiratory Chain subunits. Nucleic Acids Res..

[38] Sonney S., Leipzig J., Lott M.T., Zhang S., Procaccio V., Wallace D.C., Sondheimer N. (2017). Predicting the pathogenicity of novel variants in mitochondrial tRNA with MitoTIP. PLoS Comput. Biol..

[39] Reva B., Antipin Y., Sander C. (2011). Predicting the functional impact of protein mutations: Application to cancer genomics. Nucleic Acids Res..

[40] Herrnstadt C., Preston G., Andrews R., Chinnery P., Lightowlers R.N., Turnbull D.M., Kubacka I., Howell N. (2002). A high frequency of mtDNA polymorphisms in HeLa cell sublines. Mutat. Res..

[41] Wong L.J., Liang M.H., Kwon H., Park J., Bai R.K., Tan D.J. (2002). Comprehensive scanning of the entire mitochondrial genome for mutations. Clin. Chem..

[42] Adzhubei I., Jordan D.M., Sunyaev S.R. (2013). Predicting functional effect of human missense mutations using PolyPhen-2. Curr. Protoc. Hum. Genet..

[43] Sim N.L., Kumar P., Hu J., Henikoff S., Schneider G., Ng P.C. (2012). SIFT web server: Predicting effects of amino acid substitutions on proteins. Nucleic Acids Res..

[44] Zhai K., Chang L., Zhang Q., Liu B., Wu Y. (2011). Mitochondrial C150T polymorphism increases the risk of cervical cancer and HPV infection. Mitochondrion.

[45] Covarrubias D., Bai R.K., Wong L.C., Leal S.M. (2008). Mitochondrial DNA variant interactions modify breast cancer risk. J. Hum. Genet..

[46] Booker L.M., Habermacher G.M., Jessie B.C., Sun Q.C., Baumann A.K., Amin M., Lim S.D., Fernandez-Golarz C., Lyles R.H., Brown M.D. (2006). North American white mitochondrial haplogroups in prostate and renal cancer. J. Urol..

[47] Ebner S., Lang R., Mueller E.E., Eder W., Oeller M., Moser A., Koller J., Paulweber B., Mayr J.A., Sperl W. (2011). Mitochondrial haplogroups, control region polymorphisms and malignant melanoma: A study in middle European Caucasians. PLoS ONE.

[48] Lauber J., Marsac C., Kadenbach B., Seibel P. (1991). Mutations in mitochondrial tRNA genes: A frequent cause of neuromuscular diseases. Nucleic Acids Res..

[49] Pulkes T., Sweeney M.G., Hanna M.G. (2000). Increased risk of stroke in patients with the A12308G polymorphism in mitochondria. Lancet.

[50] Merante F., Tein I., Benson L., Robinson B.H. (1994). Maternally inherited hypertrophic cardiomyopathy due to a novel T-to-C transition at nucleotide 9997 in the mitochondrial tRNA(glycine) gene. Am. J. Hum. Genet..

[51] Zhang J., Zhang Z.X., Du P.C., Zhou W., Wu S.D., Wang Q.L., Chen C., Shi Q., Chen C., Gao C. (2015). Analyses of the mitochondrial mutations in the Chinese patients with sporadic Creutzfeldt-Jakob disease. Eur. J. Hum. Genet..

[52] Rollins B., Martin M.V., Sequeira P.A., Moon E.A., Morgan L.Z., Watson S.J., Schatzberg A., Akil H., Myers R.M., Jones E.G. (2009). Mitochondrial variants in schizophrenia, bipolar disorder, and major depressive disorder. PLoS ONE.

[53] Aitullina A., Baumane K., Zalite S., Ranka R., Zole E., Pole I., Sepetiene S., Laganovska G., Baumanis V., Pliss L. (2013). Point mutations associated with Leber hereditary optic neuropathy in a Latvian population. Mol. Vis..

[54] Sequeira A., Martin M.V., Rollins B., Moon E.A., Bunney W.E., Macciardi F., Lupoli S., Smith E.N., Kelsoe J., Magnan C.N. (2012). Mitochondrial mutations and polymorphisms in psychiatric disorders. Front. Genet..

[55] Li Y.Y., Maisch B., Rose M.L., Hengstenberg C. (1997). Point mutations in mitochondrial DNA of patients with dilated cardiomyopathy. J. Mol. Cell Cardiol..

[56] Falkenberg M., Gustafsson C.M. (2020). Mammalian mitochondrial DNA replication and mechanisms of deletion formation. Crit. Rev. Biochem. Mol. Biol..

[57] Gustafsson C.M., Falkenberg M., Larsson N.G. (2016). Maintenance and Expression of Mammalian Mitochondrial DNA. Annu. Rev. Biochem..

[58] Shadel G.S., Clayton D.A. (1997). Mitochondrial DNA maintenance in vertebrates. Annu. Rev. Biochem..

[59] Kolesar J.E., Wang C.Y., Taguchi Y.V., Chou S.H., Kaufman B.A. (2013). Two-dimensional intact mitochondrial DNA agarose electrophoresis reveals the structural complexity of the mammalian mitochondrial genome. Nucleic Acids Res..

[60] Pohjoismaki J.L., Goffart S., Tyynismaa H., Willcox S., Ide T., Kang D., Suomalainen A., Karhunen P.J., Griffith J.D., Holt I.J. (2009). Human heart mitochondrial DNA is organized in complex catenated networks containing abundant four-way junctions and replication forks. J. Biol. Chem..

[61] Pohjoismaki J.L., Wanrooij S., Hyvarinen A.K., Goffart S., Holt I.J., Spelbrink J.N., Jacobs H.T. (2006). Alterations to the expression level of mitochondrial transcription factor A, TFAM, modify the mode of mitochondrial DNA replication in cultured human cells. Nucleic Acids Res..

[62] Goffart S., Hangas A., Pohjoismaki J.L.O. (2019). Twist and Turn-Topoisomerase Functions in Mitochondrial DNA Maintenance. Int. J. Mol. Sci..

[63] Avital G., Buchshtav M., Zhidkov I., Tuval Feder J., Dadon S., Rubin E., Glass D., Spector T.D., Mishmar D. (2012). Mitochondrial DNA heteroplasmy in diabetes and normal adults: Role of acquired and inherited mutational patterns in twins. Hum. Mol. Genet..

[64] Pereira L., Soares P., Radivojac P., Li B., Samuels D.C. (2011). Comparing phylogeny and the predicted pathogenicity of protein variations reveals equal purifying selection across the global human mtDNA diversity. Am. J. Hum. Genet..

[65] Li M., Rothwell R., Vermaat M., Wachsmuth M., Schroder R., Laros J.F., van Oven M., de Bakker P.I., Bovenberg J.A., van Duijn C.M. (2016). Transmission of human mtDNA heteroplasmy in the Genome of the Netherlands families: Support for a variable-size bottleneck. Genome Res..

[66] Pohjoismaki J.L., Goffart S., Taylor R.W., Turnbull D.M., Suomalainen A., Jacobs H.T., Karhunen P.J. (2010). Developmental and pathological changes in the human cardiac muscle mitochondrial DNA organization, replication and copy number. PLoS ONE.

[67] Peeva V., Blei D., Trombly G., Corsi S., Szukszto M.J., Rebelo-Guiomar P., Gammage P.A., Kudin A.P., Becker C., Altmuller J. (2018). Linear mitochondrial DNA is rapidly degraded by components of the replication machinery. Nat. Commun..

[68] Schmitt M.W., Kennedy S.R., Salk J.J., Fox E.J., Hiatt J.B., Loeb L.A. (2012). Detection of ultra-rare mutations by next-generation sequencing. Proc. Natl. Acad. Sci. USA.

[69] Song S., Pursell Z.F., Copeland W.C., Longley M.J., Kunkel T.A., Mathews C.K. (2005). DNA percursor asymmetries in mammalian tissue mitochondrial and possible contribution to mitochondrial mutagenesis through reduced replication fidleity. Proc. Natl. Acad. Sci. USA.

[70] Nicholls T.J., Minczuk M. (2014). In D-loop: 40 years of mitochondrial 7S DNA. Exp. Gerontol..

[71] Mi J., Tian G., Liu S., Li X., Ni T., Zhang L., Wang B. (2015). The relationship between altered mitochondrial DNA copy number and cancer risk: A meta-analysis. Sci. Rep..

[72] Gorelick A.N., Kim M., Chatila W.K., La K., Hakimi A.A., Berger M.F., Taylor B.S., Gammage P.A., Reznik E. (2021). Respiratory complex and tissue lineage drive recurrent mutations in tumour mtDNA. Nat. Metab..

[73] Huijgens A.N., Mertens H.J. (2013). Factors predicting recurrent endometrial cancer. Facts Views Vis. Obgyn..

[74] Hardarson H.A., Heidemann L.N., dePont Christensen R., Mogensen O., Jochumsen K.M. (2015). Vaginal vault recurrences of endometrial cancer in non-irradiated patients-Radiotherapy or surgery. Gynecol. Oncol. Rep..

[75] Hutt S., Tailor A., Ellis P., Michael A., Butler-Manuel S., Chatterjee J. (2019). The role of biomarkers in endometrial cancer and hyperplasia: A literature review. Acta Oncol..

[76] Rosa H.S., Ajaz S., Gnudi L., Malik A.N. (2020). A case for measuring both cellular and cell-free mitochondrial DNA as a disease biomarker in human blood. FASEB J..

[77] Afrifa J., Zhao T., Yu J. (2019). Circulating mitochondria DNA, a non-invasive cancer diagnostic biomarker candidate. Mitochondrion.

