# Supplementary material for: Identification of Somatic Mitochondrial DNA Mutations, Heteroplasmy, and Increased Levels of Catenanes in Tumor Specimens Obtained from Three Endometrial Cancer Patients
Source: Life (Basel). 2022 Apr 9;12(4):562. doi: 10.3390/life12040562 (PMC9030153; doi:10.3390/life12040562)
Supplement: Supplementary file 1 [file life-12-00562-s001.zip › Supplementary.Figure S1.mjy.pdf]

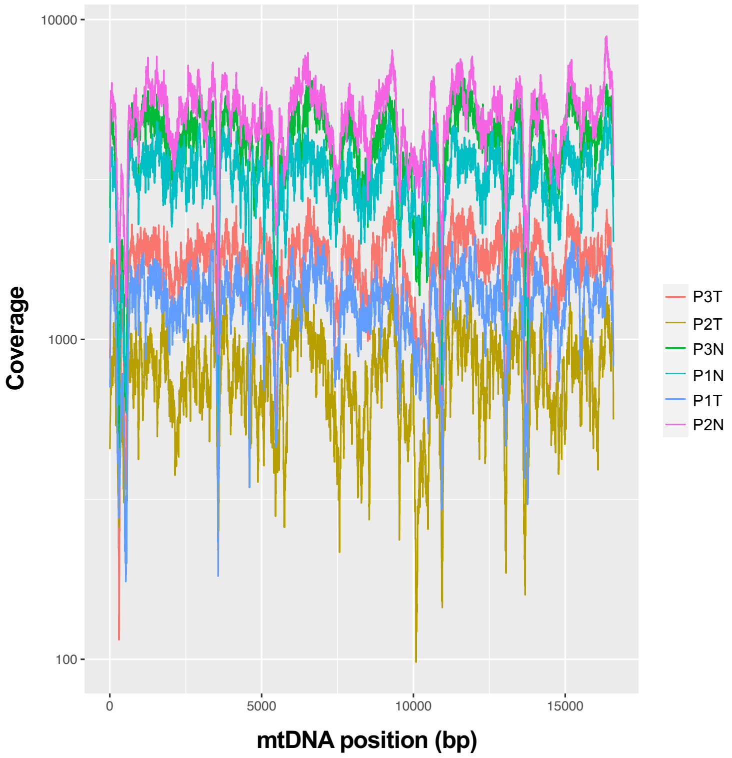

Supplementary Figure 1. Mseek deep sequencing read depth (y-axis) versus position on mtDNA (x-axis). Patients 1, 2, and 3 are P1, P2, and P3, respectively; N, perinormal tissue; T, endometrial carcinoma tumor tissue.
